# Enhanced anti-tumor activity of the Multi-Leu peptide PACE4 inhibitor transformed into an albumin-bound tumor-targeting prodrug

**DOI:** 10.1038/s41598-018-37568-6

**Published:** 2019-02-14

**Authors:** Anna Kwiatkowska, Frédéric Couture, Samia Ait-Mohand, Roxane Desjardins, Yves L. Dory, Brigitte Guérin, Robert Day

**Affiliations:** 1 Institut de pharmacologie de Sherbrooke, Université de Sherbrooke, Sherbrooke, J1H 5N4 Canada; 2 Département de Chirurgie/Urologie, Faculté de Médecine et Sciences de la Santé, Centre Hospitalier Universitaire de Sherbrooke, Sherbrooke, J1H 5N4 Canada; 3 Département de médecine nucléaire et de radiobiologie, Faculté de Médecine et Sciences de la Santé, Centre Hospitalier Universitaire de Sherbrooke, Sherbrooke, J1H 5N4 Canada; 4 Département de Chimie, Faculté des Sciences, Universitaire de Sherbrooke, Sherbrooke, J1K 2R1 Canada

## Abstract

The proprotein convertase PACE4 has been validated as a potential target to develop new therapeutic interventions in prostate cancer (PCa). So far, the most effective compound blocking the activity of this enzyme has been designed based on the structure of a small peptide Ac-LLLLRVKR-*NH*_2_ known as the Multi-Leu (ML) peptide. Optimization of this scaffold led to the synthesis of compound C23 (Ac-[DLeu]LLLRVK-amidinobenzylamide) with a potent *in vivo* inhibitory effect on the tumor growth. However, further developments of PACE4 inhibitors may require additional improvements to counter their rapid renal clearance and to increase their tumor targeting efficiency. Herein, we explored the transformation of the ML-peptide into an albumin-binding prodrug containing a tumor specific release mechanism based on the prostate-specific antigen. Our data confirms that intravenous treatment using the ML-peptide alone has little effect on tumor growth, whereas by using the ML-prodrug in LNCaP xenograft-bearing mice it was significantly reduced. Additionally, excellent *in vivo* stability and tumor-targeting efficiency was demonstrated using a radiolabelled version of this compound. Taken together, these results provide a solid foundation for further development of targeted PACE4 inhibition in PCa.

## Introduction

Prostate cancer (PCa) is the most commonly diagnosed cancer in North American men and it ranks second in cancer-related deaths^1,2^. Despite the curability of the localized disease, 30–40% of patients suffer a recurrence leading to metastatic PCa. The first line of treatment following recurrence consists of various androgen-deprivation therapies^3^. However, when disease progresses to a castration-resistant stage, there is no effective treatment (with chemotherapy being the only option), and prognosis for the patients is generally poor. Studies focusing on androgen independent pathways responsible for PCa progression may provide new therapeutic options.

The proprotein convertases (PCs) are a family of serine endoproteases that have long been associated with cancer progression because of their ability to process and activate cancer-associated substrates, for example, metalloproteinases, growth factors and their receptors^4,5^. In regards to PCa, one member of PC family, namely PACE4, has received much attention due to its overexpression in this disease state and its demonstrated role in cancer cell proliferation and tumor development^6–8^. Although this enzyme shares similar cleavage preferences for multibasic sequences Arg-X-(Arg/Lys)-Arg ↓ (X – any amino acid residue, except for Cys)^9,10^ with six other members of PC family (PC1/3, PC2, furin, PC4, PC5/6, and PC7), studies from our group have demonstrated its non-redundant function in cancer cell proliferation, tumor growth and neovascularization^6,7^. More recently, we identified an intracellular isoform of PACE4, named PACE4-altCT, that is responsible for most of tumor-cell growth capabilities and the posttranslational processing of pro-growth differentiation factor (pro-GDF15) as a first identified specific PACE4 substrate in PCa^11^. This data confirmed our previous hypothesis that PACE4 inhibitors have to penetrate cells to exert their biological effects^12^. On the other hand, the tight correlation of the PACE4-altCT overexpression and the tumor Gleason score (indicating aggressive malignancy) has been demonstrated^11^, strengthening the position of PACE4 as a new target for therapeutic drug development for PCa.

Based on the results from PACE4 silencing studies that block the tumor development in xenograft mouse models of PCa^6,7^, we developed a potent inhibitor known as the Multi-Leu (ML) peptide with the following sequence: Ac-LLLLRVKR-*NH*_2_^12^. The ML-peptide inhibits PACE4 with K_i_ value of 22 nM and exhibits potent anti-proliferative effect on PCa cell lines (DU145, LNCaP)^12^, however it only works *in vivo* if injected directly at the tumor site, whereas its intravenous administration is poorly effective^13^. This is due to both rapid clearance and poor stability. To enhance the stability profile of ML-peptide, an unnatural DLeu residue and an arginine mimetic (4-amidinobenzylamide, Amba) were introduced into its *N*- and *C*-terminal ends^14^. The resulting analog, known as compound C23 is not only more stable but also displays improved anti-cell proliferation properties *in vitro* and anti-tumor activity *in vivo*^13,15^. Its systemic administration (single dose of 2 mg/kg/day) significantly inhibits tumor progression in LNCaP xenograft model of PCa^13^. However, the same studies revealed that compound C23 is still rapidly eliminated by the kidney and has relatively short *in vivo* half-life (t_1/2_) of 9 ± 3 min^13^.

While several studies aimed at improving proteolytic stability of peptide-based leads have been proven to be effective (e.g. cyclization, chemical modifications with unnatural amino acids or peptidomimetics)^16,17^ and have been successfully applied for compound C23^15,18^, the reduction of its rapid renal clearance remains a challenge. The small size of peptides (<5 kDa) is directly responsible for their fast elimination by glomerular filtration; therefore, approaches relying on the increase of their molecular weight have been widely investigated. The most popular among them are the conjugation to large polymers, plasma proteins with long t_1/2_ or the use of the albumin binding molecules^17,19^. In regards to the ML-peptide and C23, we have already examined two strategies namely the incorporation of a lipid group or the linkage to polyethylene glycol (PEG)^15^. Both tested methods yield unsatisfactory results since the lipidation significantly increased toxicity, whereas PEGylation abolished anti-proliferative activity of the resulting analogs^15^. Therefore, we decided to turn our attention to the covalent conjugation of developed inhibitors to the albumin, which can serve as a drug carrier.

Albumin (67 kDa) is the most abundant protein in the plasma and displays characteristically long circulation t_1/2_ of 19 days in humans^20^. Due to the multiple hydrophobic binding pockets, it serves as a transporter of different ligands including fatty acids, steroids, small compounds, peptides and peptide-fatty acid chimeras specifically developed to extend the residence time of potential drugs^20–23^. In addition to the use of noncovalent interaction, the free thiol group on Cys at the position 34 of an albumin can be exploited for the chemical conjugation of various molecules^24^. Thiol–maleimide chemistry has proved to be an effective method to selectively attach the potential drugs to the albumin. As a result of this reaction, a covalent bond is formed. As a consequence, the incorporation of the release mechanism is required. In previous studies, significant progress in this area was made through the development of acid sensitive or cleavable peptide linkers that have been introduced in-between doxorubicin and an albumin-binding moiety^25–27^. The most advanced prodrug generated so far namely DOXO-EMCH (Aldoxorubicin) has reached the clinical development (Phase IIb study) exhibiting superior efficacy over doxorubicin by improving tumour response and prolonging progression-free survival for patients with advanced or metastatic soft-tissue sarcoma^28^. Of note, albumin uptake by cancer cells has been also demonstrated and used as means to transport potential drugs formulated, e.g. as the albumin nanoparticles^29,30^.

In the present study, we adapted the prodrug strategy developed by Kratz and co-workers^26,31^ and generated a tumor specific delivery system for our PACE4 inhibitor. To test the potential of this approach we chose the unmodified ML-peptide with a short *ex vivo* plasma t_1/2_ of 7.8 ± 2.3 min (compared to the t_1/2_ of our lead compound C23 of 1.74 ± 0.06 h; see Supplementary Fig. S1). The conjugation to albumin should increase the bioavailability of the ML-peptide by reducing its rate of clearance and its proteolytic degradation through the shielding effect of the nearby protein. As a release mechanism, we incorporated at the *N*-terminal position of the ML-peptide a 7-mer peptide sequence (Arg-Ser-Ser-Tyr-Tyr ↓ Ser-Leu, where ↓ indicates the cleavage site) that can be specifically recognized by prostate-specific antigen (PSA)^32^. PSA is an enzyme belonging to the kallikrein-related peptidase family of serine proteases and is secreted at low levels by normal prostatic glandular cells but is highly upregulated in prostate cancer cells^33^. Furthermore, the high levels of enzymatically active PSA are almost exclusively present in the extracellular fluid surrounding PCa sites^34^, whereas in circulation it binds to protease inhibitors and remains mostly in an inactive form^35,36^. This feature makes it an attractive candidate for targeted delivery resulting in the controlled drug release at the tumour site. Herein, we present the development of the first ML-prodrug, its cleavage profile as well as the *in vitro* and *in vivo* evaluation in the comparison to the free peptide inhibitor.

## Results

### Design of a tumor-specific delivery system for a ML inhibitor

**Figure 1:**
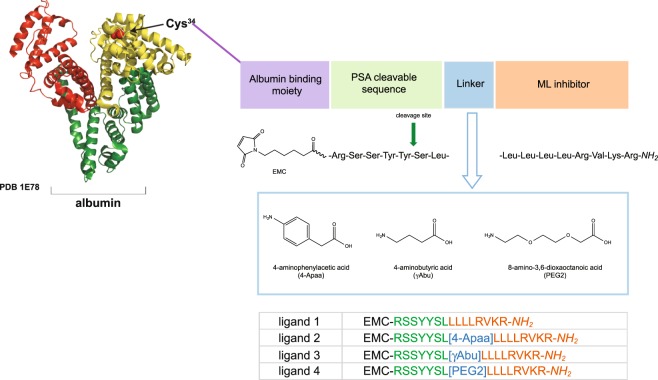
A general strategy to design ML prodrugs. The scaffold of PSA-cleavable prodrug with the highlighted specific building blocks: (1) albumin, (2) albumin binding moiety – EMC, (3) PSA-specific sequence, (4) selected linker, and (5) ML inhibitor. The chemical structures of linkers and the amino acid sequences of the ligands used in this study.

The first designed albumin binding ML-ligand was composed by the linking of three fragments via amide bonds: (i) 6-maleimidohexanoic acid (EMC) as a thiol binding moiety, (ii) PSA cleavable peptide sequence (RSSYYSL), (iii) a ML inhibitor (Fig. 1) and was synthesized using solid phase peptide synthesis (SPPS) as described in Methods. To confirm its binding affinity to albumin, the ML-ligand was incubated with the mouse serum albumin (MSA) and the formation of prodrug 1 (MSA-EMC-RSSYYSLLLLLRVKR-*NH*_2_) was monitored by a reverse-phase high-performance liquid chromatography (RP-HPLC) and matrix assisted laser desorption ionization-time of flight mass spectrometry (MALDI-TOF); for details see Methods. After 1 hour, the ligand was fully conjugated to MSA. Next, the efficiency of the release of the ML derivative SLLLLLRVKR-*NH*_2_ (peptide **1**) by human PSA (*h*PSA) was tested using prodrug **1**. The cleavage process was monitored upon the incubation of prodrug **1** with an enzymatically active *h*PSA by RP-HPLC using a synthetic peptide **1** as a control. However, even with the extended incubation time (up to 24 h) no release product was detected. We hypothesized that the lack of the PSA cleavage (not observed in the case of doxorubicin prodrugs^26,32^) might be due to the steric hindrance imposed by the ML peptide and/or reduced substrate specificity for this enzyme due to the presence of a mulit-Leu core. To address this problem, we decided to separate both peptides, i.e. a PSA-recognition sequence and a ML inhibitor by incorporating an additional linker (Fig. 1). Three linkers with different chemical properties that can be easily incorporated into a peptide structure were selected for this purpose, (*i*) an aromatic 4-aminophenylacetic acid (4-Apaa), (*ii*) a short aliphatic 4-aminobutyric acid (γAbu), and (*iii*) a short hydrophilic PEG 8-amino-3,6-dioxaoctanoic acid (PEG2). New variants of an albumin binding ligands containing selected linkers and the respective cleavage products were synthesized using SPPS (Fig. 1, Supplementary Table 1S) to perform binding and cleavage studies.

### Binding and PSA-cleavage studies

**Figure 2:**
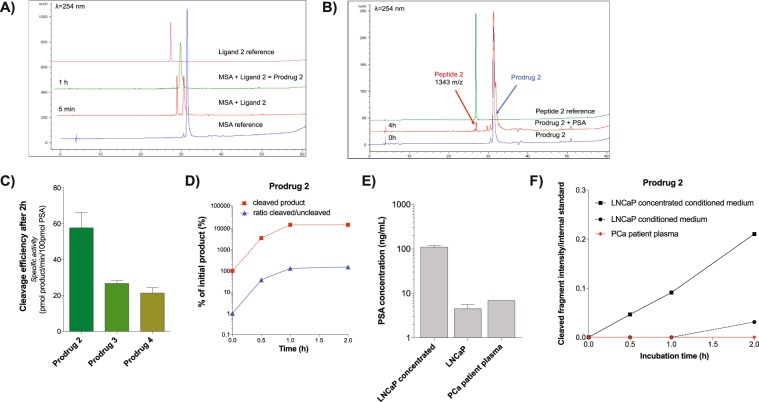
Binding and PSA-cleavage studies of ML prodrugs. (**A**) Chromatograms of incubation studies of ligand **2** with MSA. A time-course experiment showed that the reaction was completed after 1 h. (**B**) Chromatograms of *h*PSA-cleavage studies. The presence of the released compound (peptide **2**) was confirmed by RP-HPLC and MALDI-TOF analysis. (**C**) PSA-cleavage efficiency after 2 h incubation with prodrugs **2**–**4**. Prodrug 2 showed the highest conversion rate. (**D**) Release kinetic studies of prodrug 2. (**E**) PSA concentration in the selected biological samples. (**F**) The cleavage profile of prodrug **2** in different biological samples. The amount of released product (peptide **2**) correlates directly with the quantity of the enzymatically active PSA.

All three new ligands bound MSA efficiently within 1 h and were transformed into corresponding ML-prodrugs, as shown in the example of ligand 2 (Fig. 2A). In regards to the cleavage profile, PSA was able to release *N*-terminally modified ML inhibitors (peptides **2**–**4**) from all the new conjugates (chromatograms of incubation studies of prodrug **2** are presented in Fig. 2B), indicating that the incorporation of a linker (i.e. 4-Apaa, γAbu, PEG2) between the PSA substrate and the ML-inhibitor was crucial for its proteolytic action. However, the efficiency of this process was strongly influenced by the structure of the linker. As shown in Fig. 2C, prodrug **2** having a rigid 4-Apaa residue displayed considerably higher release profile in comparison to other conjugates after 2 h of incubation and this correlation remained constant even over an extended time period (see Supplementary Fig. 2S). Simultaneously, release kinetic studies of the ML-prodrug **2** were performed to determine the rate of conversion. The results showed that the highest release of a product is after 1 h of incubation with *h*PSA and the ratio of cleaved/uncleaved product remains constant for another hour (Fig. 2D). To further investigate whether prodrug **2** is also effectively cleaved in more complex biological samples and to confirm that this process is PSA specific, the release profiles were determined using different conditions in terms of enzyme concentration or its activity, such as conditioned medium from LNCaP (concentrated and not) and plasma from PCa patients (Fig. 2E). The obtained results clearly demonstrate that in this time-frame the release of the product strictly correlates with the levels of enzymatically active PSA (Fig. 2F). As expected, no product was cleaved upon incubation with plasma from PCa patients since the circulating PSA remains enzymatically inactive in the circulation^37^, whereas in the case of LNCaP concentrated conditioned medium, the highest conversion was observed.

### Activity profile of the released PACE4 inhibitors

**Figure 3:**
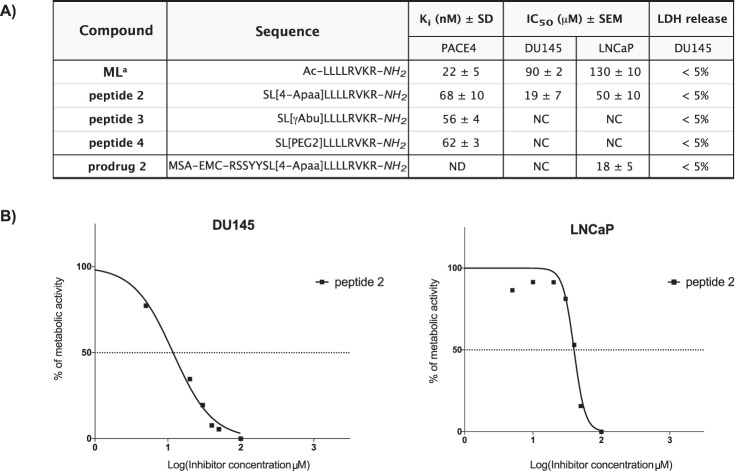
Inhibitory activity of the released analogs and prodrug 2 in comparison to ML inhibitor. (**A**) The potency of compounds against recombinant PACE4 (K_i_ values) and PCa cell lines (IC_50_ values) as well as LDH release percentage as a measure of analogs’ acute cytotoxicity. The inhibitory constant (Ki) was calculated from the IC_50_ values using the equation of Cheng and Prusoff ^50^. Data are the mean ± SD of three independent experiments. The concentration of peptides that inhibited cell growth by 50% (IC_50_) was determined by MTT cell survival assays in DU145 and LNCaP cell lines. Data represent the mean ± SEM of three independent experiments. Acute cytotoxicity of the compounds at the selected concentrations was measured by lactate dehydrogenase (LDH) assay after 4 h of incubation with DU145 cells. NC – not calculable, indicates that the curve did not converged to 50% with doses up to 300 μM; ND – not determined. (**B**) Dose−response curves for the anti-proliferative effect of peptide 2 in DU145 and LNCaP cells. Previously published compound: [a] ref.^12^. ML inhibitor was used as control and was re-assayed in presented experiments. The obtained values are comparable to previously reported.

After PSA-cleavage, we expect that the *N*-terminally modified version of the ML peptide would be released from the prodrug at the tumor site. Based on our previous results showing that even a slight change in this part of PACE4 inhibitor can completely abolish the anti-proliferative effect of the resulting analogs^15^, the activity profiles of all the new ML-peptide derivatives (peptides **2**–**4**) were assessed. As summarized in Fig. 3A, the released compounds displayed slightly lower affinity towards recombinant human PACE4 (approximately 2.5 to 3-fold) when compared to the ML peptide with the inhibition constant (K_i_) values between 56 and 68 nM. In regards to the anti-proliferative effect, only one analog containing 4-Apaa residue (peptide **2**) was able to inhibit cell growth and its activity was even improved in comparison to the ML inhibitor (with IC_50_ values of 19 ± 7 μM and 50 ± 10 μM for DU145 and LNCaP, respectively; Fig. 3A,B). The remaining peptides **3** and **4** were inactive in this cell assay. To ensure that the observed effect is due to the anti-proliferative activity rather than the acute cytotoxicity of the peptides, we measured the release of the lactate dehydrogenase (LDH), which is a marker of cell integrity. All compounds showed no acute toxicity (<5% LDH release relative to the untreated cells) after 4 h incubation with DU145 cells at the IC_50_ concentration (peptide **2**) or at 100 μM (peptides **3** and **4**). Additionally, we tested if prodrug **2** can inhibit the growth of PCa cells. The activity of this conjugate should depend on PSA-cleavage efficiency releasing the active peptide **2**; therefore, the anti-proliferative effect of prodrug **2** was evaluated in two types of cells expressing and not producing PSA (LNCaP and DU145, respectively). As shown in Fig. 3A, the tested compound was only active against LNCaP cells (with IC_50_ value of 18 ± 5 μM), indicating that PSA-cleavage is essential to induce the inhibitory effect. Given the promising overall profile of prodrug **2**, it was selected for further *in vivo* evaluation.

### Pharmacokinetic profile of prodrug 2

**Figure 4:**
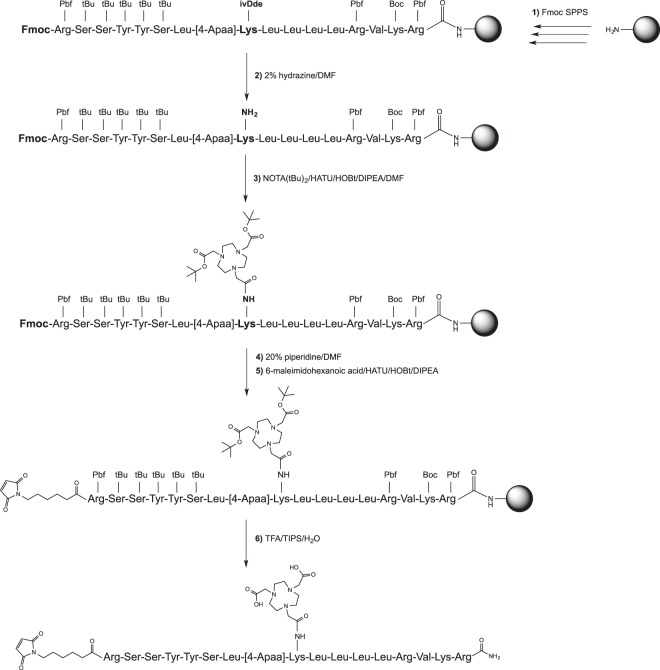
Synthetic procedure to prepare an albumin binding NOTA conjugate probe of prodrug 2. (1) Fmoc-SPPS, TentaGelS RAM resin, couplings with the protected amino acid or Fmoc-(4-Apaa)-OH (3 equiv), HATU (3 equiv) or PyBOP (3 equiv), HOBt (3 equiv) and DIPEA (9 equiv) in the mixture of DMF/DCM, 3 h in room temp.; (**2)** Selective deprotection of the ivDde group with 2%hydrazine in DMF (5×) for 5 min. (**3)** Coupling of NOTA(tBu)_2_ with HATU (3 equiv), HOBt (3 equiv) and DIPEA (9 equiv) in anhydrous DMF, o/n in room temp.; (**4)** Deprotection of the *N*-terminal Fmoc group with 20% piperidine in DMF (2×) for 10 min; (**5)** Coupling of 6-maleimidohexanoic acid (EMC) with HATU (3 equiv), HOBt (3 equiv) and DIPEA (9 equiv) in the mixture of DMF/DCM, 3 h in room temp; (**6)** Cleavage from the resin and removal of the protecting groups with TFA/TIS/H_2_O (95:2.5:2.5, v/v/v), 3 h, precipitation in cold diethyl ether, purification by semipreparative HPLC.

**Figure 5:**
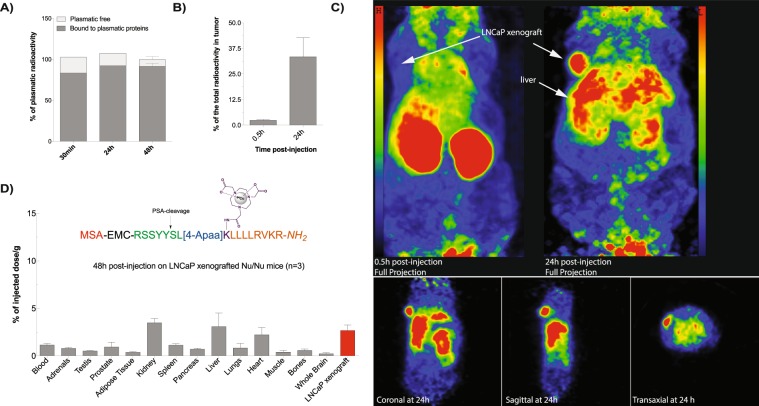
Pharmacokinetic profile of the NOTA-conjugate PET probe deriving from prodrug 2. (**A**) Distribution of the radioactivity in plasma samples collected from LNCaP xenografted mice at the selected time points. (**B**) PET-derived quantitative analyses of tumor uptake at the selected time points expressed as percentage of the remaining radioactivity. (**C**) PET images of LNCaP tumor bearing athymic nude mice at 30 min and 24 h post-injection. The images were recorded at 30 min and 24 h following the injection of radiolabeled probe. In the case of 24 h post-injection the three-dimensional projection (coronal, sagittal, and transaxial) is also presented. The white arrow indicates the xenograft location on the coronal, sagittal and transaxial images. The rest of the high signal being originating from the liver. (**D**) Post-mortem biodistribution profile of ^64^Cu-conjugated probe expressed as the percentage of the initially injected dose per gram of tissue after 48 h post-injection corrected for decay. Data are mean ± SEM (n = 3).

As the primary goal of the conjugation to albumin consists of preserving the inhibitory compound in the circulation to maintain a sustained PACE4 inhibition *in vivo*, the pharmacokinetic and tumor-targeting properties of prodrug **2** were examined. As such, prodrug **2** was converted into a molecular imaging probe that was further used to perform positron emission tomography (PET) and *ex vivo* biodistribution studies in mice bearing LNCaP tumors. In order to follow the signal coming from the released inhibitor and to ensure the optimal performance of the developed probe, the 1,4,7-triazacyclononane-triacetic acid (NOTA) bifunctional chelator was introduced on a Lys side chain between the 4-Apaa linker and the ML-peptide (far from the PSA-cleavage site and PACE4 recognition sequence; Figs 4 and 5) for ^64^Cu-radiolabeling and PET imaging. The synthesis of the probe is presented in Fig. 4 and its characterization is shown in Supplementary Table 1S. Prior to the MSA-binding, the obtained compound was radiolabeled with ^64^Cu according to the previously described protocol^38^.

Based on the previous reports showing that thiol-maleimide bond is susceptible to hydrolysis and exchange reactions with reactive thiols (e.g. from albumin, free cysteines or glutathiones) *in vivo*^39–41^, the stability of our conjugate was examined after intravenous injection by measuring the total protein bound radioactivity in plasma samples collected from mice at different time points. As shown in Fig. 5A, the majority of the administered dose (monitored as radioactivity) was detected in plasma proteins, whereas only a small proportion remained as unbound form. Additionally, the amount of the free form decreased over time (estimated as 10–35%/mL for 30 min and 1.5 ± 0.2%/mL for 48 h) suggesting it readily stayed bound to proteins in these conditions. No free ^64^Cu was detected in serum confirming its previously reported strong interactions with the NOTA chelator^42^.

Next, we investigated whether our radiolabelled-prodrug was able to reach its target once administered intravenously to LNCaP xenografted Nu/Nu mice. The results clearly showed that the peptide accumulates in the tumor in a time-dependent manner (Fig. 5B,C). After 30 min, the LNCaP tumors cumulated 2.5 ± 0.5% of the circulating radioactivity which increased to 33.5 ± 16% after 24 h (Fig. 5B), indicating an efficient tumor-specific release of the modified PACE4 inhibitor (SL[4-Apaa]K(^64^Cu/NOTA)LLLLRVKR-*NH*_2_) as a part of the albumin-bound probe and a great stability of the injected conjugate in circulation. This is easily visualized by comparing reconstructed full body PET images (Fig. 5C) where at 30 min post-injection, the activity is mostly present in highly-vascularized organs (kidney, liver, heart, and lungs) probably due to the circulating probe as well as the rapid renal elimination of the portion of unbound form characterized by the relatively small size (2.6 kDa, MW = 2606.12 g/mol). After 24 h post-injection, a very high tumor/background ratio is observed further encompassing the specific and high tumor uptake of the labelled peptide from the circulating moiety.

After 48 h post-injection the mice were euthanized and organs were harvested to be individually counted in a gamma-counter for the terminal biodistribution analysis. The results are presented as a percentage of injected doses per weight of tissue (%ID/g). As illustrated in Fig. 5D, after this relatively extended period (allowing the establishment of a steady-state level), similar percentages of the initial dose were detected in the LNCaP tumors (2.7 ± 0.9%/g), kidney (3.5 ± 0.9%/g), liver (3.1 ± 2.4%/g), and heart (2.5 ± 1.3%/g), indicating the long circulation time leading to high stability of the developed probe. For comparison, the radiolabeled version of our lead peptidomimetic compound C23 is rapidly eliminated after only 20 min post-injection^13^.

### ML prodrug has potent inhibitory effect *in vivo*

**Figure 6:**
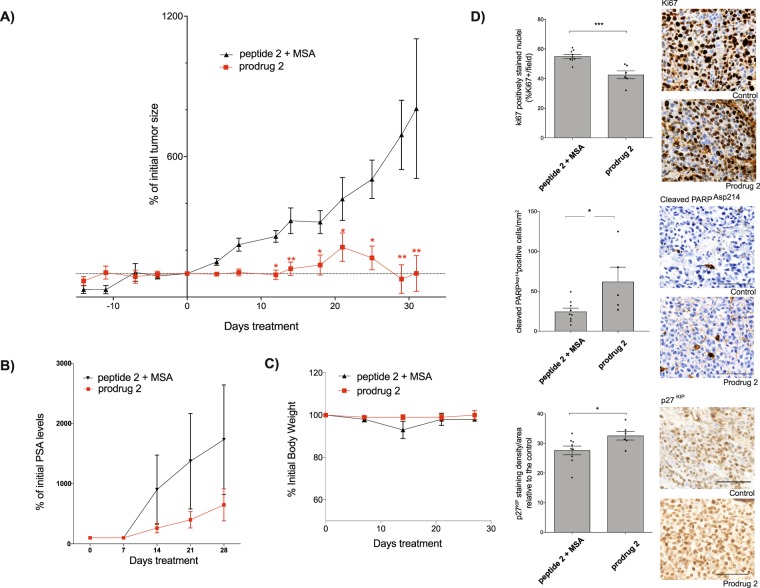
Antitumor activity of the albumin-bound prodrug 2 in comparison to the released form. (**A**) Effect of systemically administered prodrug **2** on LNCaP xenograft progression in comparison to the control group. Mice were treated with 1 mg/kg of either prodrug **2** or a synthetic peptide **2** + equimolar amount of MSA every second day intravenously. Tumor volume was measured at indicated in the time points (every 3–4 days). Data are the means ± SEM (n = 6 mice). Statistical significance was established from unpaired two-tailed student *t* tests. *p < 0.05; **p < 0.01. (**B**) Plasma PSA levels during the treatment for both groups relative to the initially measured levels. PSA levels were measured every 7 days by ELISA. (**C**) Body weight of mice during the experiment monitored every 7 days. (**D**) IHC quantitative analyzes on xenograft tissue sections. Representative images of Ki67 (cell proliferation marker), PARP (cell apoptosis marker) and p27^KIP^ (cell quiescence marker) and quantification of the respective stained tumor sections plotted as means ± SEM (n = 9 and 6 respectively for control-treated and prodrug **2** group) for both groups are shown; *p < 0.05; ***p < 0.001.

Our pharmacokinetic and biodistribution analysis reveal improved tumor targeting efficacy and stability of prodrug **2** when compared to compound C23^13^. Thus, we decided to modify our previously used protocol for *in vivo* activity evaluation in mice bearing LNCaP tumors by reducing the intravenously administrated dose (from 2 mg/kg to 1 mg/kg) and increasing the time between injections (from every 24 h to every 48 h). The mice treated with the synthetic released form (peptide **2**) in combination with an equimolar amount of MSA served as a control group. Tumor progression was monitored by measuring both tumor volumes and PSA plasmatic levels (Fig. 6A,B). The results clearly demonstrate the potent inhibitory effect of prodrug **2** on xenograft growth. As shown in Fig. 6A, when compared to the control group, the tumor progression rate is significantly lower in the group of mice treated with the prodrug **2**, which is coherent with the observed decrease in circulating PSA levels being a direct marker of LNCaP xenograft size^43^ (Fig. 6B). In regards to the toxicity of the treatment determined by the weekly body weight measurements, no weight loss was observed for the group treated with prodrug **2** (Fig. 6C).

After the completion of the treatment (14 days of tumor growth time +31 days of treatment period), LNCaP tumors were isolated from each mouse and analyzed by immunohistochemistry (IHC) for cellular proliferation, quiescence and apoptosis markers (Fig. 6D). In regards to Ki67 staining, as a marker of proliferation, our data demonstrated that the treatment with prodrug **2** led to a significant reduction (approximately 13%) in Ki67-positive cells relative to levels in tumors from the control group. Based on the cleaved PARP^Asp14^ staining that monitors cell apoptosis, a significantly higher rate of apoptosis was observed in tumors treated with prodrug **2** in comparison to the control group (approximately 37% increase, Fig. 6D). Additionally, p27^KIP^ staining indicated augmented entry into cell quiescence, as the number of p27^KIP^-postive cells were higher in tumors from the prodrug **2** group (approximately 5%) when compared to the control group. The IHC results indicate that the observed tumor growth inhibition during the treatment with prodrug **2** is caused by the reduced cell proliferation and increased cell apoptosis and quiescence, confirming the critical role of PACE4 in tumor progression.

## Discussion

Since the development of the ML-peptide, a compound with a promising *in vivo* anti-tumour activity profile^13^, its structure has been extensively studied in order to increase its stability^18^, selectivity^44,45^ and potency^15^, with excellent results that lead to its improved version known as compound C23. Even though these studies were crucial to obtain an analog with desired pharmacological effect, they might still be insufficient to translate this work into the clinic considering that compound C23 is still a small molecule that is rapidly eliminated. Therefore, we explore a prodrug approach as an efficient delivery method to the target site in order to enhance the therapeutic potential of PACE4 inhibitors.

Our delivery system is designed based on the previous work on doxorubicin^26,31^ exploiting an extremely long circulation time of an albumin as a drug-carrier, but also includes a drug-release mechanism based on the properties of an active PSA at the tumour target site. The structure of our prodrug has been optimized to include the efficient release through PSA activity, which is only possible when an appropriate linker separates the two peptides; i.e. a PSA-recognition sequence and a ML-peptide inhibitor (Figs 1 and 2). A direct linkage of these two building blocks (prodrug **1**) was not sufficient to generate a functional conjugate, highlighting the necessity to adjust the design approach for peptide-based molecules. Among all the conjugates tested, the prodrug modified with the rigid 4-Apaa (prodrug **2**) exhibited the best PSA-conversion rate. The released ML-peptide analog is *N*-terminally modified with SL[4-Apaa] fragment (peptide **2**), which may be a concern regarding its potency towards PACE4 target. However, the data clearly showed that the resulting compound displays improved activity against PCa cell lines (Fig. 3), suggesting its increased stability, since the 4-Apaa moiety acts as an unnatural residue that protects it from *N*-terminal degradation. This result is similar to our previous studies on ML-peptide analogs, with *N*-terminal modifications, such as compound C23, that contains DLeu residue at its *N*-terminal end being a substantial stability enhancer^13,15^. The sum of these results placed prodrug **2** as our lead for further investigations.

To fully evaluate the stability profile of our lead conjugate, two different methods were tested. First, we demonstrated that prodrug **2** is not cleaved prematurely in plasma from PCa patients (Fig. 2E,F), which should directly translate into its long circulation time. Then, its high stability and tumor targeting efficiency was established by the pharmacokinetic studies with the developed PET-probe (Fig. 5). The results presented demonstrate a significant and time-dependent accumulation of the released form in tumors, confirming the potential of this approach as a targeted drug-delivery system. As expected the conjugation of the ML-peptide to the albumin significantly reduced its renal elimination rate as a considerable amount of radioactivity was detectable in tumors after 24 or even 48 h post-injection. This prolonged *in vivo* stability profile is not achievable for the unconjugated ML-peptide or even its more potent counterpart compound C23, which are both rapidly eliminated from the circulation^13,38^.

The ultimate goal of these studies was to develop a delivery system for PACE4 inhibitor that once administered would result in tumor regression. Our data demonstrated the remarkable activity of prodrug **2** to inhibit tumor growth *in vivo* following its intravenous administration at the dose 1 mg/kg every 48 h. To ensure that the observed effect is due to the albumin-binding prodrug rather than the released form by itself, the control group was treated with the synthetic peptide **2** in the combination with MSA. The statistically significant difference in the tumor size observed between both groups and the reduction in PSA levels, clearly indicate that only mice receiving prodrug **2** responded to the treatment (Fig. 6A,B). Additionally, IHC analysis supported that tumor growth inhibition occurs *via* PACE4-dependent mechanism as indicated by the reduced proliferation and increased apoptosis and quiescence (Fig. 6D). It remains to be tested whether we can obtain similar results by extending the time between the administration of prodrug **2**.

In this study, we compared the ML-peptide analog (peptide **2**) with its albumin conjugated form. The peptide alone, in this test and in our previous studies regarding the unmodified ML-peptide, has no effect *in vivo*, unless it is injected directly at the tumor site^13^. Modification of the ML-peptide into compound C23 does confer *in vivo* activity, since the resulting inhibitor C23 is more stable than the ML-peptide (Fig. 1S)^13^. However, the clearance and short *in vivo* t_1/2_ of compound C23 is equivalent to the ML-peptide^13^. Our prodrug conjugate is stable enough to reach its tumor target where the *N*-terminally modified ML-peptide is released by PSA. We have not tested a conjugate with improved activity profile such as compound C23, and it may be expected that superior results can be obtained. Other possible improvements could focus on the incorporation the modifications of the release mechanism (i.e. the recognition sequence or the linker) to cleave the drug more efficiently. On the other hand, further studies will have to establish effective dose and treatment duration in various animal models. Additionally, maximally effective therapeutic outcomes may be obtained through combined therapies based on PACE4-targeting prodrug and cytotoxic agents.

In regards to potential peptide-based drugs, the linkage to the albumin aiming at improving their *in vivo* stability by reducing rate of clearance is a commonly studied strategy for compounds targeting receptors on the cell surface, such as insulin (via non-covalent binding)^46^, truncated analog of peptide YY (through covalent binding)^47^ and glucagon-like peptide 1 analogs (via non-covalent binding)^48^. So far, three of the insulin analogs containing albumin-binding fatty acids (insulin detemir, insulin degludec and liraglutide) have been approved for clinical use^49^, highlighting the potential of this approach. The present study provides evidence that this strategy can be also easily adapted for peptide-leads aiming for intracellular targets.

In conclusion, we demonstrate that the *in vivo* profile of the ML-peptide can be improved through its transformation into albumin-binding PSA-activated prodrug. The promising overall properties of prodrug **2** comprising of its high stability, tumor-targeting efficiency and potent inhibitory effect, makes it an excellent candidate for the further development and preclinical evaluation as a novel targeted therapeutic option in PCa.

## Methods

### Synthesis

All compounds were synthesized on TentaGelS RAM resin (0.23 mmol/g) using standard 9-fluorenylmethyloxycarbonyl (Fmoc) SPPS protocols. Briefly, the couplings of 3 equiv of Fmoc-protected amino acids, Fmoc-[4-Apaa] and 6-maleimidohexanoic acid (ChemImpex, USA) were performed in the solution of dichloromethane (DCM)/N,N-dimethylformamide (DMF) (1:1, v/v) using 3 equiv of benzotriazol-1-yl-N-oxytris(pyrrolidino)-phosphonium hexafluorophosphate (PyBOP) or O-(7-Azabenzotriazole-1-yl)-1,1,3,3-tetramethyluronium hexafluorophosphate (HATU) in the presence of 3 equiv of 1-hydroxy-6-chloro-benzotriazole (6-Cl-HOBt) and 9 equiv of *N*,*N*-diisopropylethylamine (DIPEA). Fmoc groups were removed by the treatment of the resin with 20% piperidine in DMF for 10 min (2×). The peptides were cleaved from resin and deprotected using the mixture of trifluoroacetic acid (TFA): triisopropylsilane (TIS): H_2_O (95:2.5:2.5; v:v:v) at room temperature for 3 h. The solutions of the released peptides were filtered, concentrated, and precipitated in cold diethyl ether. After centrifugation, the supernatants were removed, and the crude peptides were dissolved in the mixture of water/tert-butanol (1:1; v:v) and freeze-dried to white solids. The detailed synthesis of a probe is presented in Fig. 4.

All peptides were purified by RP-HPLC and the appropriate fractions were pooled and lyophilized. According to HPLC and mass spectrometry analysis the purity of obtained peptides exceeded 97%. The analytical characterization of all the compounds is presented in Supplementary Table S1.

### Inhibitory activity

Enzyme kinetic measurements were performed with recombinant *h*PACE4 as described previously^14^. Enzyme and substrate concentrations used in the present study are presented in Supplementary Table S2.

### Cell-based assays

DU145 and LNCaP cells were maintained at low passages (below 30) and cultured in RPMI 1640 (Wisent Bioproducts, QC) with 5 and 10% fetal bovine serum, respectively. For MTT proliferation assays 3500 LNCaP (on poly-L-lysine coated wells) or 1500 DU145 cells were seeded in 96-well plates, and after 24 h, the cells were treated with various concentrations of inhibitors (from 300 to 1 μM). Next, the cells were incubated for 72 h with peptides prior to addition of MTT reagent (Sigma–Aldrich, Canada) at a final concentration of 1 mg/mL. Formazan salt was solubilized with 50 μL DMSO and the metabolic activity was normalized relatively to vehicle-treated cells (sterile bi-distilled water). IC_50_ values were determined using Prism 5.0 (GraphPad Software, USA), as previously described^12^. For LDH assay, the LDH Cytotoxicity Detection Kit (Promega, USA) was used to quantify the release of lactate dehydrogenase following membrane disruption. As described in our previous work^15^, DU145 cells were seeded at a density of 3000 cells/well and allowed to incubate for 24 h. Then, the media was subsequently replaced and cells were incubated with compounds at the selected concentration. Cells treated with 1% SDS were used as a control for maximal LDH release and further relative quantification.

### MSA conjugation assay

For the conjugation to albumin, MSA (Equitech-Bio. Inc., USA) was co-incubated with EMC-ligands (at a 1:1.5; ligand: MSA molar ratio) in PBS buffer pH = 7.4 at 37 °C for 1 h. RP-HPLC and MALDI-TOF analysis were used to confirm completion of the reaction.

### hPSA cleavage

To determine PSA cleavage efficiency, peptide (125 µM final concentration; approximately 20 µg) were incubated with a final concentration of 20 µg/mL of enzymatically active *h*PSA extracted from seminal fluid (Millipore, USA) in 50 mM Tris buffer pH 7.4. Samples were analyzed by RP-HPLC (using 35 µL of the reaction solution) by comparing the resulting cleavage product over a standard curve generated using a synthetic cleaved peptide (peptides **1**–**4**). The number of nmol of a cleaved peptide produced over time was then used to calculate the specific activity in pmol product/min/100 pmol PSA to compare the different substrate peptide between each other.

### Cleavage analysis in biological samples

Peptides (125 µM final concentration) were incubated at 37 °C for the desired time points (0, 0.5, 1, 2, 4 and 8 h) together with the biological samples to be tested. Conditioned medium was obtained by culturing cells as confluent monolayers for 24 h without serum. For concentrated medium, 5 mL of medium were concentrated to 350 µL on Amicon® Ultra centrifugal filter devices (Millipore, USA) with a 3 kDa molecular weight cut-off. Plasma from PCa patient was collected in EDTA-coated tubes prior to prostatectomy procedure, centrifuged 10 min (4 °C, 1,000 × g) and immediately aliquoted and stored at −80 °C until further uses. PSA concentrations in these preparations were determined by ELISA (ClinPro International, USA). At each time point, 10 µL of the solution was taken out and quenched with 1 µL of 3% TFA. Samples were kept frozen until being concentrated and desalted using C18 ZipTip (Millipore, USA) passing the following solutions for 10 up-and-downs [acetonitrile (ACN) 0.05% TFA, water 0.05% TFA, sample, water 0.05% TFA and ACN 0.05% TFA]. Peptide cleavage was assessed by retrieving the corresponding peptide fragment intensity by MALDI-TOF analysis on a Gold Array Protein Chip (Bio-Rad, USA). Peak intensity was normalized using matrix peaks intensity (m/z: 190). All experiments and methods were performed in accordance with relevant guidelines and regulations. Patient agreed to participate and informed consent was obtained from all subjects. The research protocol was approved by the Institutional Review Committee for the Use of Human Resected Material at the Centre Hospitalier Universitaire de Sherbrooke (approval #10–017).

### Peptide-radiolabeling

Peptide was labelled as previously described^38^. Briefly, ^64^Cu was prepared following the ^64^Ni(p,n)^64^Cu reaction using an enriched ^64^Ni target electroplated on a rhodium disk and converted to [^64^Cu]Cu[II] acetate ([^64^Cu]Cu(OAc)_2_) by dissolving the [^64^Cu]CuCl_2_ in ammonium acetate (0.1 M; pH 5.5). The peptide (5 μg) was dissolved in ammonium acetate buffer (0.1 M, pH 5.5) with [^64^Cu]Cu-(OAc)_2_ (300–370 MBq; 8–10 mCi) in a total volume of 300–350 μL. The resulting solution was incubated at 95 °C for 10 min and unchelated ^64^Cu was removed on a C18 Sep-Pack Column (Waters, USA). The peptide was collected, evaporated and counted in a Capintec radioisotope calibrator (Capintec, Inc., USA) to calculate the specific activity of the product. The resulting ^64^Cu-peptide was reconstituted in PBS at pH 7.4 and used for conjugation with MSA.

### PET-scan imaging

PET scans were performed using a LabPET8 (Gamma Medica Inc., USA) small animal scanner with an axial field of view of 7.5 cm. Xenografted Nu/Nu mice were injected with 20–25 MBq (100 μL) of radiolabelled conjugates via the caudal vein under isoflurane anesthesia. Animal temperature was stabilized using a heated bed and monitored using a rectal probe. Each animal had a 20 minutes’ dynamic scan at 30 min and 24 h post-injection. The images were reconstructed by a three-dimensional MLEM algorithm implementing an analytically derived system matrix (Selivanov 2000). Regions of interest were traced for organs of interest and the respective activity was derived and reported to the injected dose/remaining radioactivity per tissue cm^3^ for calculations. Blood samples were also taken and centrifuged to obtain plasma which was precipitated by the addition of 2.5 volumes of ACN to determine the percentage of radioactivity originating from free protein-bound peptide. 48 h post-injection mice were euthanized by CO_2_ inhalation and organs of interest were further collected, washed, weighed, and measured in a gamma-counter. The results were expressed as percentage of the injected dose per gram of tissue (%ID/g). Experiments were performed with a minimum of 3 mice.

### Mouse Xenograft Assay

Trypsin-harvested LNCaP cell suspension was mixed with equal volume of matrigel (BD Biosciences, USA) on ice and subcutaneously injected (200 µL) at 2 different sites (left/right shoulder) on Nu/Nu male mice (Charles River Laboratories, Canada) at density of 2 million cells/site. Tumors were periodically measured and the tumor volumes were calculated from the equation: (Length × width^2^)/π/6. When tumors were fully formed and palpable, mice were divided into treatment groups. Compounds were given intravenously through tail vein at interval of 48 hours. The total tumor volume per animal was normalized using the following formula: (total tumor volume/total tumor volume at day 27 × 100). Blood sampling (50–75 µL) from saphenous vein was performed weekly and plasma was stored at −80 °C for PSA serum levels determination using a PSA EIA assay (ClinPro International, USA). Mice were housed under pathogen-free conditions and manipulations were performed in a biosafety cabinet. The experimental protocols were approved by the Université de Sherbrooke Ethics Committee for Animal Care (protocol number 016-16B) and use in accordance with guidelines established by the Canadian Council on Animal Care.

### Immunohistochemistry

Upon mice sacrifice, tumors were eradicated, formalin-fixed and paraffin-embedded. IHC were performed on 5 μm sections in the Department of Pathology of the CHUS (Sherbrooke, Canada) using the standard streptavidin-biotin-peroxidase immunostaining procedure with a DAKO autostainer and the solvent-resistant DAB Map detection kit (Agilent Technologies, USA). Antibodies (p27^KIP^ 1:100, PARP 1:200) were purchased from Dako Canada (Canada) or Cell Signaling Technology (USA), respectively. The ready-to-use solutions (Ki67) was obtained from Dako Canada (Canada). All slides were scanned in bright field imaging mode with a high-resolution slide scanner 223 (Nanozoomer, Hamamatsu Corporation, USA). Pictures were then analysed with NDP view 2 (Hamamatsu software). For cleaved PARP^Asp214^, IHC were conducted as described previously^11^.

Ki67 and p27 staining index were determined by manually counting up to five representative fields containing an average of 150 cells. Cells with positive nuclear staining were reported on the total number of cells per field. For cleaved PARP^Asp214^, since the number of positive cells per field was rather low (on average 1–3 per 150 cells), all positive cells were counted in as many representative tumor zones (excluding necrotic zones) and reported over the total area (in mm^2^) used for counting (calculated using Nanozoomer Digital Pathology software).

## Supplementary information

Supplementary Dataset 1
