# Supplementary material for: Enhanced anti-tumor activity of the Multi-Leu peptide PACE4 inhibitor transformed into an albumin-bound tumor-targeting prodrug
Source: Sci Rep. 2019 Feb 14;9:2118. doi: 10.1038/s41598-018-37568-6 (PMC6376031; doi:10.1038/s41598-018-37568-6)
Supplement: Supplementary file 1 — Supplementary Dataset 1 [file 41598_2018_37568_MOESM1_ESM.docx]

Supplementary data

**Enhanced anti-tumor activity of the Multi-Leu peptide PACE4 inhibitor transformed into an albumin-bound tumor-targeting prodrug.**

Anna Kwiatkowska^1,2,+,^*, Frédéric Couture^1,2,+^, Samia Ait-Mohand^1,3^, Roxane Desjardins^1,2^, Yves L. Dory^1,4^, Brigitte Guérin^1,3^, Robert Day^1,2,^*

+Authors contributed equally to this work

* [anna.kwiatkowska@usherbrooke.ca](mailto:anna.kwiatkowska@usherbrooke.ca) and robert.day@usherbrooke.ca

**Author’s affiliations:**

^1^ Institut de pharmacologie de Sherbrooke, Université de Sherbrooke, Sherbrooke, J1H 5N4, Canada

^2^ Département de Chirurgie/Urologie, Faculté de Médecine et Sciences de la Santé, Centre Hospitalier Universitaire de Sherbrooke, Sherbrooke, J1H 5N4, Canada

^3^ Département de médecine nucléaire et de radiobiologie, Faculté de Médecine et Sciences de la Santé, Centre Hospitalier Universitaire de Sherbrooke, Sherbrooke, J1H 5N4, Canada

^4^ Département de Chimie, Faculté des Sciences, Universitaire de Sherbrooke, Sherbrooke, J1K 2R1, Canada

**Table of contents**

**Figure S1.** Plasma *ex vivo* stability profile of the ML and C23 inhibitor determined by Ultra Performance Liquid Chromatography (UPLC).

**Table S1.** Analytical characterization of compounds.

**Figure S2.** Time-dependent PSA-cleavage efficiency.

**Table S2**. Enzyme and substrate concentrations used in the present study.

**Figure 1S. Plasma *ex vivo* stability profile of the ML and C23 inhibitor determined by Ultra Performance Liquid Chromatography (UPLC).** Compounds were incubated at 37°C in mouse plasma at different time points, and their plasma half-life (t_1/2_) of 7.8 ± 2.3 min for the ML inhibitor and t_1/2_ of 1.74 ± 0.06 h for compound C23 were calculated using UPLC-MS as previously described^1^.

**Table 1.** **Analytical characterization of compounds.**

| **Name** | **Sequence** | **HPLC**  [min]^a^ | **MS^c^** | |
| --- | --- | --- | --- | --- |
|  |  |  | Calcul. | Found  [M + H]+ |
| Ligand 1 | EMC-RSSYYSLLLLLRVKR-*NH_2_* | 23.46 | 2059.49 | 2061 |
| Peptide 1 | SLLLLLRVKR-*NH_2_* | 18.78 | 1209.60 | 1209 |
| Ligand 2 | EMC-RSSYYSL[4-Apaa]LLLLRVKR-*NH_2_* | 25.68 | 2192.64 | 2193 |
| Peptide 2 | SL[4-Apaa]LLLLRVKR-*NH_2_* | 22.88 | 1342.75 | 1343 |
| Ligand 3 | EMC-RSSYYSL[γAbu]LLLLRVKR-*NH_2_* | 24.10 | 2144.60 | 2144 |
| Peptide 3 | SL[γAbu]LLLLRVKR-*NH_2_* | 20.57 | 1294.70 | 1295 |
| Ligand 4 | EMC-RSSYYSL[PEG2]LLLLRVKR-*NH_2_* | 29.57 | 2204.65 | 2205 |
| Peptide 4 | SL[PEG2]LLLLRVKR-*NH_2_* | 20.68 | 1354.75 | 1355 |
| NOTA-probe | EMC-RSSYYSL[4-Apaa]K(NOTA)LLLLRVKR-*NH_2_* | 21.97^b^ | 2606.12 | 2607 |

^a^ *linear gradient from 10 to 70% of [B] in [A], ^b^ linear gradient from 25 to 50 % of [B] in [A], AGILENT Eclipse XDB C18 column; [A] 0.1% aqueous trifluoroacetic acid (TFA), [B] acetonitrile: 0.1% aqueous TFA. ^c^The molecular masses of compounds were determined with a surface-enhanced laser desorption ionisation – time of flight mass spectrometry (SELDI-TOF MS; Bio-Rad Laboratories, Hercules, CA, USA).*

**Figure 2S. Time-dependent PSA-cleavage efficiency.** The prodrugs **2** – **4** were incubated with *h*PSA at indicated time points and analyzed by HPLC and MALDI-TOF to quantify the amount of the released products.

**Table S2. Enzyme and substrate concentrations used in the present study.**

| **Enzyme** | **E_0_^a^[nM]** | **Substrate [μM]** | **K_M_^b^ [μM]** |
| --- | --- | --- | --- |
| **hPACE4** | 20.18 | 100 | 4.035 |

*^a^ The used enzyme concentrations in the assay correspond to 2 units of enzyme at the indicated substrate concentration; the enzyme concentrations in the assay (E_0_) were obtained from active-site titration using the inhibitor Dec-Arg-Val-Lys-Arg-CMK. ^b^ The K_M_ value was determined in an independent experiment with various substrate concentrations.*

**Supplemental References:**

1. Lepek, T. *et al.* Macrocyclization of a potent PACE4 inhibitor: Benefits and limitations. *Eur J Cell Biol* **96**, 476-485, doi:10.1016/j.ejcb.2017.04.001 (2017).
